# Gay and bisexual adolescents and other men who have sex with men: intersectionality and the PrEP care continuum

**DOI:** 10.11606/s1518-8787.2024058005705

**Published:** 2024-10-11

**Authors:** Eduardo Araújo de Oliveira, Lorruan Alves dos Santos, Eliana Miura Zucchi, Paula Massa, Alexandre Grangeiro, Marcia Thereza Couto

**Affiliations:** I Universidade de São Paulo. Faculdade de Medicina. Departamento de Medicina Preventiva. São Paulo, SP, Brazil Universidade de São Paulo Faculdade de Medicina Departamento de Medicina Preventiva São Paulo SP Brazil; II Universidade Católica de Santos. Programa de Pós-Graduação em Saúde Coletiva. Santos, SP, Brazil Universidade Católica de Santos Programa de Pós-Graduação em Saúde Coletiva Santos SP Brazil

**Keywords:** Adolescent, Young, Health Personnel, HIV Prevention, Intersectional Framework

## Abstract

**OBJECTIVE:**

To analyze the impact of intersecting systems of oppression on the continuum of PrEP care among adolescent gays, bisexuals, and other men who have sex with men (aGBMSM), and to examine how health professionals (HP) identify and address these challenges to provide sexual health care and HIV prevention.

**METHODS:**

This qualitative exploratory study was part of a cohort research project involving aGBMSM, *travesties*, and transgender women (aTrTW) using PrEP. Data analyzed consisted of 16 interviews with aGBMSM and eight with health professionals (HPs) in São Paulo study site. The methodological and theoretical framework for the interactive categorization of thematic analysis was based on an intersectional approach.

**RESULTS:**

The knowledge of aGBMSM about PrEP was influenced by the adverse effects of systems of oppression, particularly among Black adolescents, who acquired knowledge in a less technical manner compared to White adolescents. Most professionals recognized oppression and its impact on the PrEP care continuum (PrEPCC), especially noting the presence of racism. However, few articulated how different social markers compound barriers to the success of the PrEPCC.

**CONCLUSION:**

Social oppression affects the success of the PrEP care continuum (PrEPCC) in multiple ways. Health professionals (HPs) play a crucial role in mitigating and not perpetuating these negative experiences within health services, as well as in PrEP access, use, and adherence.

## INTRODUCTION

Despite the incorporation of new HIV prevention technologies into Brazil’s Unified Health System (SUS), such as pre-exposure prophylaxis (PrEP), the incidence of HIV has increased among gays, bisexuals, and other men who have sex with men (aGBMSM), particularly among young people and adolescents from sexual and gender minorities^1^^), (^^2^. In response to this situation, the first multicenter demonstration cohort study was initiated in 2018 to estimate the effectiveness of daily oral PrEP among aGBMSM, *travesties*, and transgender women (TrTW) aged 15 to 19 years in three Brazilian capitals^3^. The results of this study led to the incorporation of PrEP into HIV prevention policies for adolescents, as outlined in the Ministry of Health’s “Clinical Protocol and Therapeutic Guidelines for PrEP”^4^ at the end of 2022.

Despite the increase in the research on the experiences of PrEP use among young people and adolescents in Brazil^5^^), (^^6^^), (^^7^^), (^^8^^), (^^9^, there are no national studies that comprehensively articulate user and health professional perspectives on the PrEP care continuum (PrEPCC).

Drawing from the concept of the HIV prevention continuum, the PrEPCC elucidates factors that adversely affect individual and collective experiences in accessing HIV prevention methods and strategies^10^. The PrEPCC comprises several stages: quality of information received, experiences in healthcare settings, access to PrEP prescriptions, and receipt of adequate resources to maintain adherence to prophylaxis. Its relevance lies in reinforcing the understanding that the success of PrEP hinges on ensuring these stages. Moreover, the PrEPCC aims, in a dual approach, to comprehend the specificities and challenges inherent to each stage-seeking, accessing, and adhering to PrEP-and acknowledges a continuum of influences that either facilitate or hinder the prophylaxis’s success among certain groups, situations, and historical contexts.

This proposal for the use of the PrEPCC is informed by an appreciation of the complexity of personal experiences intersecting with various socio-structural factors across these three stages. Given the scarcity of studies on the PrEPCC that integrate the impact of programmatic, structural, legal, and rights frameworks into their analytical models^11^, we adopt an intersectional theoretical-methodological perspective. This approach serves as a framework to explore how these actions and frameworks influence both individual and societal levels of integrated prevention efforts.

Intersectionality focuses on understanding the interrelated processes that mutually create inequality^12^. It also facilitates examining how social structures and institutional dynamics work together to produce systemic advantages and privileges^13^. Increasingly prominent in the fields of health^14^^), (^^15^ and HIV prevention^16^^), (^^17^, intersectionality highlights the interactions among various axes of oppression such as homophobia, racism, and classism, among others, and their impacts on experiences of discrimination and stigma associated with HIV. It underscores that these intersections give rise to unique forms of inequality^18^. Therefore, intersectionality has the potential to illuminate the complex and dynamic nature of the PrEP care continuum (PrEPCC).

Based on this context, this article aims to analyze the impact of intersecting systems of oppression on the PrEP care continuum (PrEPCC) from the perspective of adolescent gays, bisexuals, and other men who have sex with men (aGBMSM). It explores how these challenges are identified and addressed by health professionals (HPs) in the context of sexual health care and HIV prevention.

## METHODS

A qualitative investigation nested in PrEP1519^3^ study was conducted to explore access to services and linkage to care within the context of community interventions aimed at creating demand for PrEP and combination prevention in the cities of São Paulo and Salvador. This analysis is based on data collected in São Paulo, involving 16 aGBMSM and eight health professionals (HPs) working at a Testing and Counseling Center (TCC) participating in the demonstration study.

The criteria for the participation of aGBMSM using PrEP in the qualitative research were as follows: 1. Having used PrEP for at least one month; 2. Diversity in terms of demand creation strategies to access the PrEP service; 3. Diversity in terms of place of residence, ethnicity/skin color, and educational level; and 4. Diversity of experiences with PrEP use, including participants with good adherence, participants facing challenges in using the medication or in clinical follow-up, and participants who discontinued PrEP use.

Using a pre-tested interview guide, in-depth interviews were conducted with 16 aGBMSM from 2019 to 2020 and with eight HPs in 2020. The interview guide covered themes such as: knowledge about the prophylaxis; barriers and facilitators to initiating and adhering to PrEP; experiences with STI testing services; use of preventive methods; and aspects related to ethnicity, gender, sexuality, and socioeconomic backgrounds. The guide used for the interviews with HPs focused on: the management of offering and monitoring PrEP use; the dynamics of interactions between aGBMSM and healthcare professionals and services; and experiences in handling situations of violence experienced by adolescents.

The interviews with adolescents were conducted face-to-face at locations chosen by the participants, such as healthcare facilities, their homes, or NGOs they attended. The interviews with HPs were conducted via video call platforms due to the restrictions imposed by the COVID-19 pandemic. All interviews were recorded, transcribed, anonymized, and carefully reviewed to ensure content quality.

In the analysis and interpretation, we utilized thematic interactive categorization^19^ with intersectional sensitivity^14^, drawing from Hancock’s framework^20^. This approach posits that intersectional empirical analyses should encompass the pertinent social markers that arise from empirical data and the variable relationships among them. According to this perspective, intersections among social markers are not merely additive, and the significance of each cannot be predetermined, as they collectively shape distinct dynamics of vulnerability and privilege^18^. The analysis also considered the interplay between individual, institutional, and structural factors.

The stages of analysis included: conducting the interviews; defining the analytical categories by the research team and extracting empirical data accordingly; describing the emergence of social markers within these categories, emphasizing their interconnectedness and the parameters of advantages and disadvantages they influence; developing a preliminary synthesis for each analytical category, along with formulating hypotheses supported by relevant literature; and conducting a final synthesis by comparing the preliminary findings with existing literature on the subject of investigation.

PrEP1519 Study was approved by the Ethics committees of the institutions involved (number 89993018.9.0000.0065) and was conducted in accordance with the resolutions of the Brazilian National Health Council (Resolutions 466/2012 and 510/2016). Participants aged 18 and above provided signed informed consent. Adolescents aged 15 to 17 signed an informed assent form, which was granted by a judicial authorization that waived parental consent.

## RESULTS

### Sociodemographic characterization

The age of the aGBMSM interviewed ranged from 17 to 20 years old, with eleven being Black and five White. Fifteen participants lived with their parents or relatives, while one lived alone. Regarding sexual orientation, thirteen identified as gay, one as pansexual, and two as bisexual (see Table 1). Among the eight HPs interviewed, four were Black and four were White. Their ages ranged from 25 to 52 years old. Two identified as heterosexual and six as gay. Three professionals were psychologists (two worked as counselors in the PrEP1519 study and one as a liaison), three were medical doctors, one was a biomedical doctor (serving as an assistant coordinator), and one was a nursing technician. The average duration of employment in the PrEP1519 study was 12.5 months (see Table 2).

**Table 1:** Sociodemographic characterization of the aGBMSM

Pseudonym	Age (years)	Skin Color	Sexual Orientation	Gender Identity	Education Level	Duration of PrEP use
Benjamin	18	Black	Gay	Cisgender man	Complete high school	9 months
Bernardo	17	White	Gay	Cisgender man	Attending high school	3 months
Fernando	17	White	Gay	Cisgender man	Attending high school	2 months
Hugo	20	Black	Gay	Cisgender man	Attending high school	2 months
Jackson	17	Black	Pansexual	Cisgender man	Attending high school	2 months
Jonathan	19	Black	Gay	Cisgender man	Attending university	3 months
Nicolau	17	Black	Gay	Cisgender man	Attending high school	1 month
Jorge	18	Black	Gay	Cisgender man	Attending university	2 months
Pedro	19	White	Gay	Cisgender man	Attending university	2 months
Theo	18	White	Bisexual	Cisgender man	Attending university	3 months
Oscar	20	Black	Gay	Cisgender man	Attending university	6 months
Miguel	20	Black	Gay	Cisgender man	Attending university	5 months
Luiz	20	Black	Bisexual	Cisgender man	Attending university	8 months
Juliano	20	White	Gay	Cisgender man	Incomplete university	4 months
Renato	19	Black	Gay	Cisgender man	Complete high school	1 month
Thiago	18	Black	Gay	Cisgender man	Attending high school	6 months

**Table 2:** Sociodemographic characterization of the health professionals

Pseudonym	Age (years)	Skin Color	Sexual Orientation	Gender Identity	Background	Role in the PrEP 1519 project	Time working in the PrEP 519 study
Valentina	43	Black	Heterosexual	Cisgender woman	Psychology	Counselor in charge of participants’ retention in care	12 months
Sofia	52	White	Heterosexual	Cisgender woman	Biomedicine	Health service coordination assistant	8 months
Romeo	29	White	Gay	Cisgender man	Psychology	Counselor in charge of participants’ retention in care	4 months
Matteo	35	Black	Gay	Cisgender man	Medicine	Physician	6 months
Enzo	27	White	Gay	Cisgender man	Medicine	Physician	3 months
Enrico	25	Black	Gay	Cisgender man	Nursing technician	Nursing technician	21 months
Antonela	34	White	Gay	Cisgender woman	Medicine	Physician	27 months
Dante	26	Black	Gay	Cisgender man	Psychology	Counselor in charge of participants’ linkage to care	19 months

### Previous trajectories of the aGBMSM in HIV prevention

The aGBMSM showed diversity in their prior HIV prevention methods before PrEP, including condom use, HIV post-exposure prophylaxis (PEP), and the use of lubricant gel. Failures and inconsistencies were reported, such as issues with condoms, such as erection loss, forgetfulness, not having the product available during sex, and challenges in negotiating use with partners.

The adolescents’ experiences in seeking and using health services-specifically for general health and STI testing-revealed three distinct groups: the first group consisted of adolescents who relied on their parents, predominantly their mothers, to schedule and attend appointments (two aged 17, two aged 20, and one aged 18). The second group included those who never depended on their parents or caregivers for these actions (one aged 16, two aged 19, two aged 20, one aged 18, and three aged 17). The third group comprised adolescents who, while maintaining connections with caregivers for general health consultations, independently sought and managed their sexual health care (two adolescents, one aged 19 and the other aged 18). All adolescents expressed feelings of insecurity, fear, and concern regarding HIV testing, regardless of whether they were accompanied, familiar with the service, or had previous testing experience. These fears were rooted in past sexual exposure.

### The effect of the intersection of systems of oppression on the PrEP care continuum

Adolescents justified their motivations for seeking PrEP as stemming from sexual experiences without condoms, whether due to personal preference, difficulty in using them, or objections from their sexual partners. Additionally, within the context of psychoactive substance use, seeking PrEP provided a sense of safety similar to that associated with condoms.

The axes of social oppression impacted various aspects of the lives of aGBMSM, particularly their sexual experiences and their approaches to HIV/STI prevention. The three stages of the PrEP care continuum (PrEPCC) - knowledge, access, and adherence to PrEP - were intersected by these axes, especially influenced by the effects of racism, classism, and ableism.

There were gaps in access to knowledge about PrEP, particularly reported by black adolescents who learned about the prophylaxis from sexual partners during face-to-face encounters or through dating apps. They perceived the information as incomplete, especially regarding the methods and frequency of pill usage, and had concerns about managing potential adverse effects. Consequently, these adolescents sought additional information on the internet and in sexual health services. For instance, Benjamin, an 18-year-old Black adolescent with a high school education, mentioned that despite frequent visits to a sexual health service for HIV/STI testing, he never received any information about PrEP from the clinic. He only became aware of the prophylaxis through internet searches. In contrast, Hugo, a 20-year-old White university student, learned about PrEP during classes conducted by a health professional specializing in the subject.

Racism acts as a barrier by constraining access to health services and denying rights to aGBMSM. Benjamin’s account reveals how intersecting axes of oppression operate structurally and institutionally. In contrast, Theo, an 18-year-old bisexual White individual, did not perceive his skin color as a hindrance in establishing social relationships or in accessing and using PrEP. Additionally, his privileged social status, exemplified by having medical insurance, amplified the disparities in experiences and privileges compared to Black adolescents.

Before this year [before turning 18], it was my parents. Then, as of this year, I have had more freedom to use the health insurance card and I am the one who schedules appointments with my doctors. [...] I do not think that this [skin color] affects my life at all (Theo).

[...] Because, mainly, some public policies do not include this [Black] population. This population is usually socially invisible and cannot access a number of things that a middle-class person who lives in another environment or has a different perspective can access [...] I consider myself privileged, because I can access places that most people like me have never accessed (Benjamin).

The interaction between skin color and social class showed that Benjamin, a Black man with moderate economic means, had relatively unrestricted social mobility and therefore access to various social spaces, including health services. However, racism manifested in other areas of his life, such as experiencing harassment from a security guard in a market.

Other systems of social oppression, such as ableism, have intensified oppression and adversely affected access to PrEP knowledge and services, as well as the overall sexual experience. Oscar, a 20-year-old Black man with visual impairment, recounted instances where people expressed surprise or astonishment at his presence in social settings. He also mentioned never receiving information about PrEP during multiple visits to the HIV testing service until he encountered one of the healthcare professionals (HPs) involved in the PrEP1519 study.

### The management of structural oppression by health professionals and PrEP research

HPs are expected to identify and address barriers within the PrEP care continuum (PrEPCC). The coordinator of the PrEP1519 study facilitated interface with services, enhancing training, supporting improved care workflows, managing complex situations, and fostering integration within the service network. Consequently, it is assumed that the professionals interviewed were aware in identifying instances of racism, ableism, classism, and homophobia within the PrEPCC and the lives of adolescents. The supportive working conditions, which included training, discussions on complex cases, and supervision, enabled HPs to critically examine the stark disparities between low-income black adolescents and middle-class white adolescents, as highlighted by Dante, a psychologist/counsellor. For Dante, the focus was not on privileging Black teenagers over White ones but on recognizing the systemic advantages afforded to White individuals while acknowledging the disadvantages faced by Black individuals. Like Dante, the other professionals most commonly identified barriers to accessing health services in the accounts of Black adolescents, such as financial constraints, geographical distance from services, and subjective concerns such as fear of facing police violence while heading to the service.

Based on the reports shared by the adolescents during their follow-up appointments, the multidisciplinary team reviewed the care processes and procedures to strengthen adolescents’ engagement with the service. Several strategies were highlighted, including: maintaining contact with adolescents between appointments through text messages and phone calls; and establishing mechanisms to follow-up adolescents during their journey from home to the health service, addressing insecurities and fears while fostering a sense of support throughout the process.

[...] So, I think it is essential to try to understand where this person is speaking from, and I often say that my listening is different when I am talking to a Black *travesti* or a Black boy. These people feel and are more vulnerable [...] (Dante).

The collective decision of the HPs to identify and address the impacts of social oppression in the daily lives of adolescents did not entirely prevent situations of discomfort. Matteo, a Black cisgender man, shared his struggles in providing assistance to non-binary individuals. Despite his extensive knowledge of gender and sexuality, societal norms around cisgender identity interfered with his ability to assist effectively, leading to challenges and discomfort in using gender-neutral pronouns. This difficulty could potentially affect the adherence and retention of this social group.

Gender neutral pronouns. It is something that makes me go “Hmm...” But I confess that sometimes it comes out [the use of pronouns according to the cisnormative reference], right? On this point, I confess that I police myself more (Matteo).

While professionals demonstrated sensitivity to various forms of social oppression overall, attention to racism was particularly notable in the narratives of Black professionals. It was also evident that, among the stages of the PrEP care continuum (PrEPCC), access was perceived as the most affected. Racism was recognized as a barrier to effective mobility in the city, whether via public transport or on foot, thereby impacting the ability of aGBMSM to reach services.

[...] So, we come across young people who find it hard to leave the house, right? There are young Black people, too, because of being Black. So, it is difficult to go to the subway, it is hard, you know? [...] (Valentina).

Dante, a Black psychologist/counsellor, and Valentina, a Black psychologist/representative, acknowledged the intersection of oppressive axes as creators of vulnerabilities that necessitate specific approaches to listening and guidance.

[...] A White cis boy is different from a Black cis boy. I remember when I talked to a Black cis boy and he said: “Look, I wish I was more of a bottom, but I am more of a top. People think that just because I am a Black man I have to play this role.” And then I told him that no, this role can be developed if he wants, but he can engage in other sexual practices that he enjoys [...] (Dante).

Finally, HPs perceived the discontinuity in PrEP use among aGBMSM as stemming from vulnerabilities such as family conflicts, discrimination, and prejudice. While HPs expressed intent to address some of these issues, they recognized the impossibility for the service and the team to fully manage the complexity surrounding the subjective and structural factors contributing to PrEP interruptions and potential HIV infections.

## DISCUSSION

The findings highlight the direct and indirect impact of intersecting systems of social oppression, particularly emphasizing racism and classism experiences across the stages of the PrEP care continuum (PrEPCC).

The participants in this study had either completed or partially completed high school (8) and university (8), indicating a higher level of education compared to the general population. However, their profile was similar to the overall profile of current PrEP users in Brazil^21^.

Intersectional axes of oppression result in disparate experiences of the stages of the PrEP care continuum (PrEPCC) among individuals from marginalized social groups and those with societal privileges. Similar findings were reported in other studies examining how HIV/STI prevention experiences are adversely affected by social oppression axes^5^^), (^^20^. Racism has historically impacted HIV prevention efforts - initially through stigmatizing associations between the infection and black Haitian men^22^, and, currently, due to the challenges faced by Black men in accessing and participating in preventive strategies^23^.

A recent Brazilian study conducted with young people from sexual minorities using PrEP^5^ indicated that Black and economically disadvantaged individuals faced greater challenges in accessing knowledge about and adhering to the prophylaxis, due to the intersection of race and class. Similarly, a study involving young Black individuals in Toronto, Canada, utilized intersectionality as a theoretical and methodological framework to analyze the impact of race and class on the stigma surrounding PrEP and barriers to accessing information about it^24^. The literature supports the findings discussed in the interviews with Hugo and Benjamin: Hugo learned about PrEP from a lecturer during his medical studies, whereas Benjamin found out about it in casual dating apps. This illustrates how structural racism helps Black individuals from actively participating in academic spheres and contributes to their potential alienation from scientific discussions on PrEP and HIV prevention methods, leading to gaps in knowledge and a reliance on settings that may lack comprehensive information and scientific evidence.

Our findings also highlight how lesser-discussed axes of oppression, such as ableism, constrain the full expression of sexuality and hinder access to PrEP. For instance, Oscar, a Black adolescent with a disability, expressed feeling less desirable among his peers in places of intimate and sexual encounters. This underscores the importance of theoretical insights into the social factors influencing the expression of desire and the societal construction of desirable bodies.

Another aspect of this issue pertains to how healthcare professionals perceive these individuals and their vulnerability to HIV. The account from one participant, who were sexually active, regularly visited the health service for HIV testing, yet never received information about PrEP, illustrates how the intersection of ableism with other axes of oppression negatively impacted the PrEP care continuum (PrEPCC), particularly in terms of information and access. The professionals’ and services’ failure to acknowledge the participant’s active sexual life due to his visual impairment hindered his access to preventive measures. To reach health services, Oscar had to navigate a lengthy and arduous journey in a city not adapted to his condition.

The way in which healthcare professionals (HPs) operate, ideally aimed at mitigating disparities in access to healthcare rights of this nature, underscores the significant role of institutional barriers in perpetuating inequality in sexual health care for aGBMSM. This participant with visual impairment had a low level of education, a history of STIs, and made frequent visits to health services; yet, he had uncertainties about preventive methods, only knew about PEP, and did not receive information about PrEP during these visits. The challenges adolescents face in accessing health services are profound^25^, compounded by encounters with professionals who, by not recommending PrEP use, contribute to their vulnerability to HIV infection.

The analysis concerning the healthcare professionals (HPs) reveals that, unlike many professionals working in sexual health and HIV prevention services in Brazil, these workers participated in the training and monitoring provided by the study. Their narratives are strongly aligned with human rights frameworks and focused on actions aimed at avoiding the perpetuation of social oppressions such as racism, ableism, homophobia/biphobia, and classism experienced daily by aGBMSM.

The presence of Black HPs appeared to contribute to a more nuanced understanding of the structural racism experienced by aGBMSM. In a study involving young people using PrEP, racism was identified as a barrier to continued use. Participants described the professionals as passive-aggressive; they were not explicitly racist but failed to meet Black participants at the agreed time, prioritizing White individuals instead. This contributed to a negative perception of both the HPs and the service, which did not address these forms of segregation identified by users^26^. Consistently with our findings, this study emphasizes the importance of providing care that does not reinforce the marginalization of aGBMSM.

In relation to the stages of the PrEPCC, access was identified by health professionals and users as the most affected by social oppression, primarily due to racism and homophobia. Other studies have highlighted participants’ discomfort in receiving disrespectful treatment, not only based on their skin color but also on their sexual orientation, thereby violating their rights to accessing health services^26^. Conversely, the higher proportion of Black participants in both this qualitative study and the demonstration study cohort (68%)^27^ can be viewed as an indicator of successful diversification in demand creation strategies. Thus, through a combination of community interventions at social gathering places for young people, virtual networks, and dating apps^27^, it becomes feasible to include a more diverse profile of young people and adolescents, considering their sociability dynamics and vulnerability to HIV, in the provision of PrEP.

While the stages of knowledge and access to PrEP were the most highlighted ones by both HPs and adolescents, due to the detrimental effects of social oppression, adherence did not appear to be significantly impacted. This could possibly be attributed to the strong bond established between the adolescents, the service, and the HPs.

An epidemiological study conducted with health professionals in a PrEP service indicated that skin color did not influence the prescription of the pill, in contrast to our findings which suggest that this factor can either hinder or facilitate PrEP use^28^. However, the same epidemiological study emphasized that being a man who has sex with another man is a highly relevant factor for health professionals, given the elevated rates of HIV infection within this population. This aligns with our findings that HPs are aware and proactive in addressing barriers in the PrEPCC that affect aGBMSM’s access to prophylaxis.

Some limitations of this study need to be acknowledged. Firstly, participants in our study exhibited a relatively high level of education compared to the general population of Brazilian adolescents, which aligns with the typical profile of PrEP users in the country. This could introduce bias in identifying experiences of social oppression in daily life. Additionally, the aGBMSM participants were actively engaged in regular clinical follow-up for PrEP, which might limit our insights to those individuals who have more positive evaluations of the prophylactic method, especially concerning adherence in the PrEPCC stage. Moreover, those who agreed to participate in the interviews may have been more inclined to share their experiences related to the stages of the PrEPCC, potentially excluding perspectives from those who chose not to collaborate.

## CONCLUSIONS

The significance of the findings presented here lies in the integrated analysis of perspectives from two complementary segments in PrEPCC studies: users and professionals. Intersectionality has proven to be a valuable analytical framework for understanding how the stages of the PrEPCC are influenced by interconnected axes of social oppression. Regarding the experiences shared by aGBMSM, it is evident that the acquisition of PrEP knowledge was adversely affected by racism, while other social markers such as class and disability also compounded barriers to accessing sexual health care and HIV prevention. The accounts from professionals underscored the challenges and nuanced approach required for clinical management grounded in principles that prioritize acknowledging diverse forms of social oppression and their interplay.

On the other hand, professionals reported a limited and fragmented perception and recognition of the intersecting effects of various axes of oppression. While they acknowledged the impact of racism, they did not elaborate on its connections with other axes. Given the results mentioned above, it is essential for health centers to proactively integrate actions aimed at consistently mitigating social oppression in the realm of HIV and other STI prevention.
