# Supplementary material for: Gay and bisexual adolescents and other men who have sex with men: intersectionality and the PrEP care continuum
Source: Rev Saude Publica. 2024 Oct 11;58(Suppl 1):11s. doi: 10.11606/s1518-8787.2024058005705 (PMC11573366; doi:10.11606/s1518-8787.2024058005705)
Supplement: Translation

# Translation: Adolescentes gays, bissexuais e outros homens que fazem sexo com homens: interseccionalidade e continuum de cuidado de PrEP

**Authors:** Eduardo Araújo de Oliveira, Lorruan Alves dos Santos, Eliana Miura Zucchi, Paula Massa, Alexandre Grangeiro, Marcia Thereza Couto

**Affiliations:** I Universidade de São Paulo. Faculdade de Medicina. Departamento de Medicina Preventiva. São Paulo, SP, Brasil Universidade de São Paulo Faculdade de Medicina Departamento de Medicina Preventiva São Paulo SP Brasil; II Universidade Católica de Santos. Programa de Pós-Graduação em Saúde Coletiva. Santos, SP, Brasil Universidade Católica de Santos Programa de Pós-Graduação em Saúde Coletiva Santos SP Brasil

**Keywords:** Adolescente, Jovem, Pessoal de Saúde, Prevenção ao HIV, Enquadramento Interseccional

## Abstract

**OBJETIVO:**

Analisar os impactos dos sistemas de opressão intersectados no continuum do cuidado de profilaxia pré-exposição sexual (CCPrEP) entre gays, bissexuais e outros homens que fazem sexo com homens (aGBHSH) e como estes são identificados e tratados por profissionais de saúde (PS), visando o cuidado à saúde sexual e prevenção do HIV.

**MÉTODOS:**

Estudo de investigação qualitativa integrado a uma pesquisa de coorte com aGBHSH e mulheres transexuais e travestis (aMTrT) em uso de PrEP. Foram analisados dados produzidos na cidade de São Paulo, integrando 16 entrevistas com aGBHSH e oito com PS. O referencial teórico metodológico se orientou pela categorização interativa de análise temática com enquadramento interseccional.

**RESULTADOS:**

Os conhecimentos sobre PrEP dos aGBHSH são afetados pelos efeitos negativos dos sistemas de opressão, sobretudo entre adolescentes negros que adquirem conhecimento de forma menos técnica quando comparados aos brancos. A maioria dos profissionais enxerga as opressões e as suas consequências, e confere protagonismo ao racismo, mas poucos articulam os diferentes marcadores sociais que potencializam barreiras para o sucesso do CCPrEP.

**CONCLUSÕES:**

As opressões sociais impactam o sucesso do CCPrEP de diferentes maneiras. Os PS são centrais para mitigação e não propagação dessas experiências negativas dentro do serviço de saúde e no acesso, uso e adesão à PrEP.

## INTRODUÇÃO

Apesar da incorporação de novas tecnologias de prevenção ao HIV no Sistema Único de Saúde do Brasil (SUS), como a profilaxia pré-exposição sexual (PrEP), a incidência de HIV tem crescido entre gays, bissexuais e outros homens que fazem sexo com homens (GBHSH) e, preocupantemente, entre jovens e adolescentes de minorias sexuais e de gênero^1^^), (^^2^. Diante desse cenário, em 2018, se iniciou o primeiro estudo de coorte demonstrativo multicêntrico para estimar a efetividade da PrEP oral diária entre adolescentes GBHSH e mulheres transexuais e travestis (TrMT), com idade entre 15 e 19 anos, em três capitais brasileiras^3^. Os resultados deste estudo impulsionaram, no final de 2022, a incorporação da PrEP nas políticas de enfrentamento do HIV em adolescentes, conforme o “Protocolo Clínico e Diretrizes Terapêuticas para PrEP” do Ministério da Saúde^4^.

A despeito do aumento de investigações sobre a experiência de uso de PrEP entre jovens e adolescentes no Brasil^5^^), (^^6^^), (^^7^^), (^^8^^), (^^9^, desconhecemos estudos nacionais que articulem as percepções e experiências relacionadas ao continuum do cuidado de PrEP (CCPrEP) na articulação das perspectivas dos usuários e dos profissionais de saúde.

Baseado na noção de continuum da prevenção ao HIV, o CCPrEP elucida fatores que impactam negativamente e restringem experiências individuais e coletivas de acesso aos métodos e estratégias de prevenção ao HIV^10^.

O CCPrEP inclui as seguintes etapas: qualidade do conhecimento recebido, experiências nos serviços de saúde, acesso à prescrição de PrEP e recebimento dos recursos adequados para a manutenção da adesão à profilaxia. Sua relevância se justifica na medida em que reforça a compreensão do sucesso da PrEP depender da garantia de tais etapas. Adicionalmente, o CCPrEP almeja, em um duplo movimento, entender as particularidades e desafios intrínsecos a cada etapa - busca, acesso e adesão à PrEP - e reconhecer que há um continuum de forças que atuam facilitando ou dificultando o sucesso da profilaxia em determinados grupos, situações e momentos históricos.

A proposta de utilização do CCPrEP se orienta pela apreensão da complexidade das experiências pessoais no entrecruzamento com outros fatores socioestruturais envolvidos em suas três etapas. Considerando que há poucos estudos sobre o CCPrEP que incluem a influência das ações programáticas e estruturais e dos marcos legais e de direitos nos seus modelos analíticos^11^, tomamos como referência a perspectiva teórico-metodológica da interseccionalidade para a compreensão de como tais ações e marcos afetam os níveis individuais e sociais da prevenção combinada.

A interseccionalidade concentra-se nas relações entre os processos mutuamente constituídos que criam as desigualdades^12^. Além disso, permite examinar como as estruturas sociais e as dinâmicas institucionais operam para produzir vantagens e privilégios sistêmicos^13^. Com crescente destaque no campo da saúde^14^^), (^^15^ e nos estudos em prevenção ao HIV^16^^), (^^17^, a interseccionalidade enfatiza a interação entre eixos de opressão (homofobia, racismo, classismo e outros) e seus impactos nas experiências de discriminação e estigmas associados ao HIV, bem como tem a capacidade de demonstrar que essas interseções são produtoras de desigualdades únicas^18^. A interseccionalidade, portanto, tem o potencial de explicitar a natureza complexa e dinâmica do CCPrEP.

Com base nesse panorama, o objetivo do artigo é analisar, da perspectiva de aGBHSH, os impactos dos sistemas de opressão intersectados que afetam o CCPrEP e como eles são identificados e manejados por profissionais de saúde (PS) visando o cuidado com a saúde sexual e a prevenção ao HIV.

## MÉTODOS

Realizou-se investigação qualitativa integrada ao estudo PrEP1519^3^, buscando compreender o acesso a serviços e a vinculação ao cuidado no contexto de intervenções comunitárias para criação de demanda para PrEP e prevenção combinada nas cidades de São Paulo e Salvador. A análise apresentada é baseada nos dados produzidos em São Paulo, com 16 aGBHSH e oito PS contratados para atuar, pelo estudo, em um Centro de Testagem e Aconselhamento (CTA).

A participação dos aGBHSH usuários de PrEP na investigação qualitativa seguiu os seguintes critérios: 1. estar em uso de PrEP há pelo menos um mês; 2. a diversidade em termos de estratégias de captação e criação de demanda pelas quais os aGBHSH acessaram o serviço; 3. diversidade de local de moradia, raça/cor de pele e escolaridade e; 4. diversidade de experiência de uso de PrEP, incluídos aqueles com boa adesão, com dificuldades no uso da medicação e/ou seguimento clínico e aqueles com situações de abandono.

Foram realizadas entrevistas em profundidade, com roteiro previamente testado, com 16 aGBHSH, entre 2019 e 2020, e oito PS, em 2020. O roteiro aplicado aos adolescentes explorou: conhecimento sobre a profilaxia, barreiras/facilitadores para início e permanência na PrEP, experiências nos serviços de testagem para IST, uso de métodos preventivos, além de aspectos relacionados às identidades de raça, gênero, sexualidade e classe. O roteiro aplicado aos PS explorou: manejo da oferta e acompanhamento do uso de PrEP, vínculo dos aGBHSH com os profissionais e serviços de saúde, além das experiências de manejo de outras situações de violência vividas pelos adolescentes.

As entrevistas com os adolescentes ocorreram de forma presencial, em locais de escolha dos participantes, como serviço de saúde, residência e ONG que frequentam. As entrevistas com os PS foram realizadas virtualmente, em plataforma de videochamadas, devido às restrições causadas pela pandemia de covid-19. As entrevistas foram gravadas, transcritas, anonimizadas e conferidas para assegurar a qualidade do conteúdo textual.

Na análise e interpretação, adotou-se uma categorização interativa temática^19^, com sensibilidade interseccional^14^ baseada nos pressupostos de Hancock^20^ de que análises empíricas interseccionais devem considerar os marcadores sociais relevantes que emergem do empírico e as relações variáveis entre eles. Nesses termos, as intersecções entre os marcadores sociais não constituem a simples soma de partes e nem o peso de cada um deles pode ser considerado *a priori*, pois juntos contribuem para construção de dinâmicas únicas de vulnerabilização e privilégio^18^. A análise considerou as dinâmicas entre fatores individuais, institucionais e estruturais.

As etapas de análise consistiram em: impregnação das entrevistas; definição das categorias de análise pelo grupo de pesquisadores, bom como posterior extração do material empírico; descrição de como os marcadores sociais emergiram nas referidas categorias, destacando a força de articulação entre eles segundo os parâmetros de vantagens e desvantagens que operam; construção de síntese preliminar para cada categoria de análise, em articulação com a criação de “hipóteses” amparadas na literatura sobre o tema e; síntese final com cotejamento dos achados da síntese preliminar com a literatura sobre o objeto de investigação.

O estudo PrEP1519 foi aprovado, sob o número 89993018.9.0000.0065, pelos comitês de ética das instituições envolvidas e os dados produzidos atenderam as resoluções do Conselho Nacional de Saúde do Brasil (466/2012 e 510/2016). Os participantes maiores de 18 anos assinaram o Termo de Consentimento Livre e Esclarecido. Mediante autorização judicial, adolescentes com idade entre 15 e 17 anos participaram do estudo com assinatura do Termo de Assentimento Livre e Esclarecido.

## RESULTADOS

### Caracterização sociodemográfica

A idade dos aGBHSH entrevistados variou de 17 a 20 anos, 11 eram negros e cinco brancos. 15 participantes moravam com os pais ou familiares e um sozinho. Quanto à orientação sexual, 13 se declaram homossexuais, um pansexual e dois bissexuais (Quadro 1). Dos oito PS entrevistados, quatro eram negros e quatro brancos. A idade dos participantes variou de 25 a 52 anos. Dois se identificaram como heterossexuais, e seis como homossexuais. Três profissionais tinham formação em psicologia (dois atuavam como retentores e um como vinculador), três eram médicos, uma era biomédica (atuação de assistente de coordenação) e um era técnico de enfermagem. A média de tempo de atuação no estudo PrEP1519 foi de 12,5 meses (Quadro 2).

**Quadro 1:** Caracterização sociodemográfica dos aGBHSH

Codinome	Idade (anos completos)	Cor da Pele	Orientação Sexual	Identidade de Gênero	Escolaridade	Tempo de uso de PrEP
Benjamin	18	Negro	Gay	Homem cisgênero	Ensino médio completo	9 meses
Bernardo	17	Branca	Gay	Homem cisgênero	Ensino médio cursando	3 meses
Fernando	17	Branca	Gay	Homem cisgênero	Ensino médio cursando	2 meses
Hugo	20	Negro	Gay	Homem cisgênero	Ensino médio cursando	2 meses
Jackson	17	Negro	Pansexual	Homem cisgênero	Ensino médio cursando	2 meses
Jonathan	19	Negro	Gay	Homem cisgênero	Ensino superior cursando	3 meses
Nicolau	17	Negro	Gay	Homem cisgênero	Ensino médio cursando	1 mês
Jorge	18	Negro	Gay	Homem cisgênero	Ensino superior cursando	2 meses
Pedro	19	Branca	Gay	Homem cisgênero	Ensino superior cursando	2 meses
Theo	18	Branca	Bissexual	Homem cisgênero	Ensino superior cursando	3 meses
Oscar	20	Negro	Gay	Homem cisgênero	Ensino superior cursando	6 meses
Miguel	20	Negro	Gay	Homem cisgênero	Ensino superior cursando	5 meses
Luiz	20	Negro	Bissexual	Homem cisgênero	Ensino superior cursando	8 meses
Juliano	20	Branca	Gay	Homem cisgênero	Ensino superior incompleto	4 meses
Renato	19	Negro	Gay	Homem cisgênero	Ensino médio completo	1 mês
Thiago	18	Negro	Gay	Homem cisgênero	Ensino médio cursando	6 meses

**Quadro 2:** Caracterização sociodemográfica dos profissionais de saúde

Codinome	Idade (anos completos)	Cor da Pele	Orientação Sexual	Identidade de Gênero	Formação	Atuação no Projeto PrEP 1519	Tempo de atuação no estudo PrEP 1519
Valentina	43	Negra	Heterossexual	Mulher cis	Psicóloga	Retentora	12 meses
Sofia	52	Branca	Heterossexual	Mulher cis	Biomédica	Assistente de coordenação do serviço de saúde	8 meses
Romeo	29	Branco	Homossexual	Homem cis	Psicólogo	Retentor	4 meses
Matteo	35	Negra	Homossexual	Homem cis	Médico	Médico	6 meses
Enzo	27	Branco	Homossexual	Homem cis	Médico	Médico	3 meses
Enrico	25	Negro	Homossexual	Homem cis	Técnico de enfermagem	Técnico de enfermagem	21 meses
Antonela	34	Branca	Homossexual	Mulher cis	Médica	Médica	27 meses
Dante	26	Negro	Homossexual	Homem cis	Psicólogo	Vinculador	19 meses

### Trajetórias prévias dos aGBHSH em prevenção ao HIV

Os aGBHSH apresentaram diversidade quanto às formas de prevenção ao HIV previamente à PrEP, como uso de preservativo, profilaxia pós-exposição sexual ao HIV (PEP) e gel lubrificante. Falhas e inconsistências foram relatadas, por exemplo, no caso do preservativo, devido a perda de ereção, esquecimento, não ter o insumo no momento da transa e dificuldade de negociação do uso com o parceiro.

As experiências dos adolescentes na busca e uso do serviço de saúde - em termos de saúde em geral e testagem de IST - permitiram a identificação de três grupos: o primeiro constituído pelos que dependiam dos pais, principalmente das mães, para agendamento e comparecimento aos atendimentos (dois adolescentes com 17 anos, dois com 20 anos e um com 18 anos); o segundo agregando os que nunca dependiam de pais/cuidadores para essas ações (um adolescente de 16 anos, dois com 19 anos, dois com 20 anos, um de 18 anos e três com 17 anos); e o terceiro grupo compreendido por aqueles que, embora mantivessem ligação com os seus cuidadores para consultas em saúde geral, apresentavam autonomia na busca e cuidado da saúde sexual (dois adolescentes, um com 19 e outro com 18 anos). Todos demonstraram sentimento de insegurança, medo e preocupação para realização da testagem do HIV, independentemente de estarem ou não acompanhados, conhecerem ou não o serviço ou já terem realizado testagem. Os temores se justificam pelas exposições sexuais prévias.

### O efeito da intersecção dos sistemas de opressão no continuum do cuidado de PrEP

As motivações para busca da PrEP foram justificadas pelos adolescentes em decorrência de experiências sexuais sem preservativo, fosse por preferência pessoal, dificuldade de uso ou objeção do parceiro. Adicionalmente, no contexto de uso de substâncias psicoativas, a busca por PrEP mobilizou sensação de segurança associada ao preservativo.

Os eixos de opressão social atuaram sobre diferentes aspectos das vidas dos aGBHSH, particularmente no que tange à vida sexual e à forma como pensam e fazem prevenção ao HIV/ISTs. As três etapas do CCPrEP (conhecimento, acesso e adesão à PrEP) foram afetadas por esses eixos de forma interseccionada, especialmente pela ação do racismo, classismo e capacitismo.

Lacunas no acesso ao conhecimento sobre PrEP foram relatadas sobretudo por adolescentes negros, que conheceram a profilaxia por meio de parcerias afetivossexuais, em encontros presenciais ou em aplicativos de encontro. A percepção de incompletude das informações, sobretudo acerca das modalidades e frequência de uso dos comprimidos, e as dúvidas no manejo de potenciais efeitos adversos os motivaram a buscar mais informações na internet e em serviços de saúde sexual. Benjamin, 18 anos, negro, ensino médio completo, relatou que apesar de ir com frequência ao serviço de saúde sexual para realizar testagem para HIV/IST (TS-HIV), nunca recebeu qualquer tipo de informação sobre PrEP dos PS e só soube da profilaxia na internet. Em contrapartida, Hugo, 20 anos, branco e estudante universitário, relatou ter descoberto a PrEP em aulas com profissional de saúde especialista no tema.

O racismo atua dificultando o acesso a serviços de saúde e negando direitos aos aGBHSH. O relato de Benjamim revela a articulação de eixos de opressão que operam em termos estruturais e institucionais. Theo, 18 anos, bissexual e branco, ao contrário, desconhece a sua cor da pele como fator limitante para o estabelecimento de relações sociais em geral e tampouco para seu acesso e uso da PrEP. Ademais, sua boa condição social se expressa em possuir convênio médico, o que amplifica diferenças de experiência, em termos de privilégios, em relação aos adolescentes negros.

Antes desse ano [antes de completar 18 anos] eram os meus pais. Aí, a partir desse ano, eu tenho mais liberdade pra usar o cartão do convênio e quem marca meus médicos sou eu. [...] Acho que isso [cor da pele] não afeta a minha vida, pra nada (Theo).

[...] Porque, principalmente, algumas políticas públicas não incluem essa população [negra]. E que normalmente é invisibilizada e que não consegue ter acesso a um número de coisas que uma pessoa de classe média que vive em outros ambientes ou tem outras perspectivas [...] Eu me considero privilegiado, porque eu consigo acessar lugares onde a maioria das pessoas como eu nunca acessaram (Benjamin).

A articulação entre cor da pele e classe social revelou que Benjamin, um homem negro com condição econômica razoável, consegue ter trânsito social menos restrito e, por consequência, acessar outros espaços sociais, incluindo serviços de saúde. Não obstante, para ele, o racismo se manifestou em outras esferas, como ser perseguido no mercado pelo segurança.

Outros sistemas de opressão social, como o capacitismo, incrementaram as opressões e afetaram negativamente o acesso ao conhecimento e ao serviço de PrEP, bem como a própria experiência sexual. Oscar, 20 anos, negro e com deficiência visual, descreveu o estranhamento das pessoas quanto a sua presença em locais de sociabilidade, expresso por olhares de espanto ou surpresa. Além disso, afirmou que nunca teve acesso às informações sobre PrEP nas diversas idas ao serviço para realizar TS-HIV, até ser atendido por um dos PS do estudo PrEP1519.

### Os manejos das opressões estruturais por profissionais de saúde e pesquisa PrEP

É esperado que os PS identifiquem e atuem para dirimir as barreiras para CCPrEP. A atuação da coordenação do estudo PrEP1519 na interface com o serviço agregou capacitação, no sentido de apoiar a melhoria dos fluxos de atendimento, o manejo de situações de maior complexidade e a integração com a rede de serviços. Assim, supõe-se que os profissionais entrevistados estivessem atentos à identificação de situações envolvendo racismo, capacitismo, classismo e homofobia no CCPrEP e nas vidas dos adolescentes. As condições favoráveis de trabalho, que incluíam capacitação, discussão de casos complexos e supervisão, propiciaram um olhar diferenciado dos PS para as diferenças abissais entre adolescentes negros de baixa renda e brancos de classe média, como apontado por Dante, psicólogo/vinculador. Para ele, não se tratava de privilegiar os adolescentes negros e descredibilizar os brancos, mas de reconhecer a força da estrutura social que privilegia pessoas brancas ao mesmo tempo que traz desvantagens para as negras. Dante, assim como outros dois profissionais, identificou mais comumente nas falas dos adolescentes negros as barreiras no acesso ao serviço de saúde, como falta de dinheiro, distância entre o local de moradia e o serviço, e outras condições subjetivas, a exemplo do medo de sofrer violência policial no trajeto até o serviço.

A partir dos relatos compartilhados pelos adolescentes nas consultas de acompanhamento, a equipe multiprofissional revisou processos e procedimentos do atendimento visando incrementar as chances de vinculação dos adolescentes com o serviço. Entre as estratégias, destacaram-se: realização de contatos entre as consultas via mensagens de texto e ligação; e a criação de mecanismos de acompanhamento dos adolescentes no trajeto entre o local de moradia e o serviço de saúde, para acolher inseguranças e medos e aumentar a sensação de amparo durante o deslocamento.

[...] Então acho que é essencial tentar entender de onde essa pessoa fala, e eu costumo dizer que a minha escuta é diferente quando eu tô falando com uma travesti negra ou com um menino preto. Essas pessoas se sentem e são mais vulneráveis [...] (Dante).

A decisão compartilhada dos PS em identificar e manejar as repercussões das opressões sociais no cotidiano dos adolescentes não impediu que situações limítrofes ao desconforto acontecessem. Matteo, homem cis e negro, expressou dificuldade no atendimento a pessoas não binárias. Apesar do vasto conhecimento sobre gênero e sexualidade, a “norma” social cisgênera interfere no seu atendimento, expressa no estranhamento e dificuldade na utilização do pronome neutro, o que poderia resultar em menor adesão e retenção desse grupo social.

Pronome neutro. É uma coisa que eu fico assim “Hum...” Mas eu confesso que às vezes sai [o uso dos pronomes segundo a referência cisnormativa], né? Esse ponto, eu confesso que eu me policio mais (Matteo).

Embora no conjunto das narrativas os profissionais denotem sensibilidade a diferentes opressões sociais, a atenção ao racismo se destacou principalmente pelos profissionais negros. Também foi expressivo que, entre as etapas do CCPrEP, o acesso foi visto como o mais afetado, pois o racismo é reconhecido como uma barreira para efetivação do trânsito social na cidade, via transporte público ou a pé, impactando a chegada dos aGBHSH ao serviço.

[...] Então a gente se depara com jovem que têm dificuldade de sair de casa mesmo, né? Tem jovem negro, também, pela questão de ser negro. Então é difícil ir no metrô, é difícil, né? [...] (Valentina).

Dante, psicólogo/vinculador negro, e Valentina, psicóloga/retentora negra, reconheceram a intersecção dos eixos de opressão como produtores de vulnerabilidades que necessitam de manejos específicos na escuta e nas orientações.

[...] É diferente de um menino cis branco e de menino cis negro. Eu lembro que, quando eu conversei com um menino cis negro, ele falou: “Olha eu queria ser mais passivo, mas eu sou mais ativo. As pessoas acham, só porque eu sou um homem negro, eu tenho que desenvolver esse papel”. E aí eu fui conversar com ele que não, esse papel pode ser desenvolvido caso queira, mas ele pode ter outras práticas sexuais das quais tenha prazer [...] (Dante).

Finalmente, a descontinuidade no uso da PrEP pelos aGBHSH foi percebida pelos PS como decorrente de atravessamentos de vulnerabilidades em distintas instâncias, como conflitos familiares, discriminação e preconceito. E, ainda que os PS demonstrassem intenção de lidar com alguns desses aspectos, reconheceram a impossibilidade de o serviço e a equipe “darem conta” da complexidade que envolve as dimensões subjetivas e estruturais que contribuem para a interrupção da PrEP, acarretando potencial infecção pelo HIV.

## DISCUSSÃO

Os resultados revelam a ação direta e indireta do cruzamento de diferentes sistemas de opressão social, com especial destaque para o racismo e classismo nas experiências relacionadas às etapas do CCPrEP.

Os participantes deste estudo possuem ensino médio completo ou incompleto, (8) e ensino superior completo ou incompleto (8), o que consiste em uma amostra com alta escolaridade quando comparada à população em geral, mas similar ao perfil atual dos usuários de PrEP no Brasil^21^.

Os eixos de opressão, quando interseccionados, fazem com que as etapas do CCPrEP sejam vividas de formas discrepantes entre indivíduos pertencentes às minorias sociais e aqueles com privilégios sociais. De forma similar, esses resultados também foram encontrados em outros estudos que avaliaram como a experiência da prevenção ao HIV/IST foi impactada negativamente pelos eixos de opressão social^5^^), (^^20^. A prevenção ao HIV sempre foi impactada pelo racismo - inicialmente, por haver uma correlação estigmatizante da infecção com os homens negros haitianos^22^, e, atualmente, pelas dificuldades que homens negros têm para acessar e se vincular às estratégias preventivas^23^.

Recente estudo brasileiro realizado com jovens de minorias sexuais usuários de PrEP^5^ aponta que pretos e pobres foram mais prejudicados no acesso ao conhecimento e na adesão à profilaxia, em decorrência da intersecção de classe e raça. Em estudo com jovens negros de Toronto (Canadá), a interseccionalidade também serviu como referencial teórico metodológico na análise de raça e classe na produção do estigma da PrEP e nas barreiras de acesso ao conhecimento sobre PrEP^24^. A literatura corrobora os resultados expressos nas entrevistas de Hugo e Benjamin. Hugo acessa conhecimento sobre a PrEP por meio de um professor do curso médico; Benjamin, a partir de aplicativos de relacionamentos casuais. Isso demonstra o quanto o racismo estrutural opera para dificultar que pessoas pretas possam frequentar de forma mais destacada a esfera acadêmica e o quanto essa situação pode gerar afastamento de debates científicos sobre PrEP e métodos preventivos ao HIV, contribuindo para o desconhecimento e para a necessidade de busca sobre prevenção em ambientes potencialmente precários de informações e evidências científicas.

Nossos resultados também revelam como outros eixos de opressão menos presentes na literatura sobre prevenção ao HIV, como o capacitismo, atuam de modo a restringir o exercício integral da sexualidade e o acesso à PrEP. O exemplo de Oscar, adolescente negro com deficiência que se sente menos desejável pelos pares em locais de encontros afetivossexuais aponta para a utilidade de reflexões teóricas sobre os fatores sociais que interferem na expressão desses desejos e na produção social de corpos desejáveis.

A outra faceta desse problema se refere ao modo como os PS enxergam esses indivíduos e suas vulnerabilidades ao HIV. O relato de um dos participantes de que tem uma vida sexual ativa, frequenta o serviço de saúde para realizar TS-HIV e nunca obteve qualquer informação sobre PrEP explicita como a intersecção do capacitismo com outros eixos de opressão incrementou negativamente o CCPrEP, particularmente quanto à informação e ao acesso. O relato de não reconhecimento, por parte dos profissionais e dos serviços, de sua vida sexual ativa no contexto da deficiência visual impacta seu acesso ao repertório de prevenção. Em relação ao acesso ao serviço de saúde, Oscar precisa enfrentar um longo e penoso trajeto em uma cidade não adaptada para sua condição.

A via de atuação dos PS, que idealmente deveriam mitigar disparidades de acesso a direitos em saúde dessa natureza, corrobora o peso das barreiras institucionais na (re)produção das desigualdades de cuidado com a saúde sexual de aGBHSH. O nosso participante com deficiência visual era alguém com baixo conhecimento, histórico de IST e de idas frequentes ao serviço de saúde; ele tinha dúvidas sobre métodos preventivos, conhecia somente a PEP e não teve acesso à PrEP durante esses comparecimentos. A dificuldade dos adolescentes para chegarem ao serviço é imensa^25^ e, ainda, se deparam com profissionais que ao não aconselharem o uso da PrEP colaboram para vulnerabilidade de infecção ao HIV.

As análises relativas aos PS revelam que, diferentemente de grande parte dos profissionais atuantes nos serviços de saúde sexual e em prevenção ao HIV no Brasil, eles participaram de treinamentos e monitoramentos oferecidos pelo estudo. Suas narrativas foram, na maioria das vezes, alinhadas ao referencial dos direitos humanos e às ações voltadas a não (re)produção de opressões sociais advindas do racismo, capacitismo, homofobia/bifobia e classismo que os aGBHSH vivem no cotidiano.

A composição de negros entre os PS parece ter contribuído para um olhar mais refinado para o racismo estrutural vivido pelos aGBHSH. Em um estudo realizado com jovens que utilizavam PrEP, identificou-se o racismo como fonte impeditiva para continuidade do uso. Os participantes descreveram os profissionais como passivo-agressivos, não demonstraram racismo, mas não os atendiam no horário combinado, passando pessoas brancas na frente, contribuindo para uma percepção negativa dos PS e do serviço, que se isentava nas resoluções dessas segregações identificadas pelos usuários^26^. Em concordância com nossos achados, este estudo propõe uma reflexão sobre a importância da realização de atendimentos que não promovam o incremento da marginalização de aGBHSH.

Em relação às etapas do CCPrEP, o acesso foi tido tanto pelos profissionais de saúde quanto pelos usuários como o mais prejudicado pelas opressões sociais, principalmente em decorrência do racismo e da homofobia. Outros estudos demonstraram o desconforto dos participantes em serem tratados com descaso, não somente por consequência da cor da pele, mas também por conta da orientação sexual, o que resulta em violação de direitos ao acesso de serviços de saúde^26^. Por outro lado, a maior proporção de participantes negros, tanto nesta investigação qualitativa como na coorte do estudo demonstrativo (68%)^27^, pode ser lida como um indicador de sucesso da diversificação das estratégias de criação de demanda. Assim, ao combinar intervenções comunitárias em locais de sociabilidade de jovens, redes virtuais e aplicativos de encontros^27^ torna-se possível incluir um perfil mais diversificado de jovens e adolescentes, do ponto de vista das dinâmicas de sociabilidade e de vulnerabilidade social ao HIV, na oferta de PrEP.

Enquanto as etapas de conhecimento e acesso à PrEP foram as que mais se destacaram nos depoimentos dos PS e dos adolescentes, em virtude dos prejuízos por consequência das opressões sociais, a adesão não foi uma etapa impactada de forma relevante, possivelmente pelo vínculo estabelecido entre adolescentes com o serviço e os PS.

Estudo epidemiológico realizado com profissionais de saúde em serviço de PrEP demonstrou que a cor da pele não influenciava na prescrição do comprimido, diferente do cenário do nosso estudo, que demonstra esse fator pode prejudicar ou facilitar o uso da PrEP^28^. Entretanto, o mesmo estudo epidemiológico destacou que ser um homem que faz sexo com outro homem é um atributo de alta relevância para os profissionais de saúde, dadas as taxas elevadas de infecção por HIV nessa população. Isso corrobora nossos achados de que os PS são sensibilizados para atuar nas barreiras do CCPrEP que afastam os AGBHSH do uso da profilaxia.

Algumas limitações deste estudo devem ser consideradas. Os participantes apresentam perfil de escolarização relativamente elevado, comparativamente à população de adolescentes brasileiros, embora condizente com o perfil de usuários de PrEP no país. Esse dado pode configurar viés na identificação das opressões sociais nas experiências cotidianas. Os aGBHSH estavam em acompanhamento clínico regular de PrEP, o que pode limitar nossas análises ao conjunto de pessoas com avaliações mais positivas sobre o método profilático, particularmente quanto à etapa da adesão no CCPrEP. Adicionalmente, os usuários que decidiram participar das entrevistas podem ter sido aqueles que se sentiam mais confortáveis em compartilhar as experiências relacionadas às etapas do CCPrEP, em comparação aos que não aceitaram colaborar.

## CONCLUSÕES

A relevância dos achados aqui apresentados está na análise articulada de percepções e interpretações das experiências de dois segmentos complementares nos estudos do CCPrEP, os usuários e os profissionais. A interseccionalidade se mostrou uma perspectiva útil de análise que permite compreender como as etapas do CCPrEP são afetadas pelos eixos de opressões sociais articulados. No que tange às experiências narradas pelos aGBHSH, observa-se que a aquisição do conhecimento de PrEP foi afetada negativamente pelo racismo, embora outros marcadores sociais como classe e deficiência tenham contribuído para ampliar as barreiras ao acesso e cuidado da saúde sexual e da prevenção do HIV. Nos relatos dos profissionais, foram elencadas dificuldades e sensibilidades necessárias para o manejo clínico ancorado em princípios que valorizem o reconhecimento das opressões sociais e suas dinâmicas.

Por outro lado, os profissionais relataram de forma incipiente e fragmentada a percepção e reconhecimento dos efeitos intersectados dos eixos de opressão. Embora tenham reconhecido o efeito do racismo, não explicitaram a articulação deste com outros eixos. Tendo em vista os resultados apresentados, é imprescindível que haja um movimento dos PS para incorporar de forma consistente ações de mitigação de opressões sociais no âmbito assistencial de prevenção ao HIV e outras IST.
